# Quantitative analysis of amino acid excretion by *Methanothermobacter marburgensis* under N_2_-fixing conditions

**DOI:** 10.1038/s41598-025-87686-1

**Published:** 2025-01-30

**Authors:** Barbara Reischl, Benjamin Schupp, Hayk Palabikyan, Barbara Steger-Mähnert, Christian Fink, Simon K.-M. R. Rittmann

**Affiliations:** 1 Archaea Physiology & Biotechnology Group, Department of Functional and Evolutionary Ecology, University of Vienna, Wien, Austria; 2 Arkeon GmbH, Tulln a.d. Donau, Austria; 3 BioOceanography and Marine Biology, Department of Functional and Evolutionary Ecology, University of Vienna, Wien, Austria

**Keywords:** Biotechnology, Microbiology, Archaea, Anaerobes, Methanogens, Diazotrophy, Biofuel, Bioproduct, Biorefinery, Microbiology techniques, Applied microbiology, Archaea, Environmental microbiology, Industrial microbiology, Chemical engineering

## Abstract

Methanogenic archaea (methanogens) possess fascinating metabolic characteristics, such as the ability to fix molecular nitrogen (N_2_). Methanogens are of biotechnological importance due to the ability to produce methane (CH_4_) from molecular hydrogen (H_2_) and carbon dioxide (CO_2_) and to excrete proteinogenic amino acids. This study focuses on analyzing the link between biological methanogenesis and amino acid excretion under N_2_-fixing conditions. Among five hydrogenotrophic, autotrophic methanogens, *Methanothermobacter marburgensis* was prioritized and further cultivated in closed batch cultivation mode under N_2_-fixing conditions. *M. marburgensis* was grown on chemically defined minimal medium with different concentrations of ammonium in a H_2_/CO_2_/N_2_ atmosphere. This enabled the quantification of ammonia uptake, N_2_-fixation, amino acid excretion and the conversion of H_2_/CO_2_ to CH_4_. To quantify N_2_-fixation rates in a mass balance setting a novel method has been established. The method utilizes the pressure drop below a certain threshold pressure in closed batch cultivation mode – the *th*reshold *p*ressure for *N*_*2*_*-fix*ation (THp_N2fix_). Using the THp_N2fix_ method, volumetric N_2_-fixation rates of *M. marburgensis* as high as 0.91 mmol L^−1^ h^−1^ were determined. Excretion of amino acids was found with highest detected values of glutamic acid, alanine, glycine and asparagine. The highest total amino acid excretion of 7.5 µmol L^−1^ h^−1^ was detected with H_2_/CO_2_/N_2_ at an ammonium concentration of 40 mmol L^−1^. This study sheds light on the link between methanogenesis, biological N_2_-fixation, and proteinogenic amino acid excretion. The concomitant production of amino acids and CH_4_ could become of biotechnological relevance in an integrated approach coupling biomethanation and N_2_-fixation in a biorefinery concept.

**Supplementary Information:**

The online version contains supplementary material available at 10.1038/s41598-025-87686-1.

## Introduction

The greenhouse gasses (GHGs) carbon dioxide (CO_2_), methane (CH_4_) and nitrous oxide (N_2_O) accumulate in the atmosphere of Earth where they contribute heavily to global warming and climate change. The accumulation of GHGs mainly results from anthropogenic combustion of fossil fuels, industrial processes and agriculture^1–3^. One example for high CO_2_ emissions in industrial processes is the production of ammonia via the Haber-Bosch process. The Haber-Bosch process catalyzes the reaction of molecular hydrogen (H_2_) and molecular nitrogen (N_2_) to ammonia and demands a metal catalyst, high temperature (400–500 °C) and high pressure (150–350 bar) and therefore results in 1.5 tons CO_2_ per ton NH_3_ produced^4–6^. From a nitrogen perspective, 174 million tons of NH_3_ are produced each year globally applied as artificial fertilizers to ensure an increased harvest of crops^4,7^. Thus, biological nitrogen N_2_-fixation (diazotrophy), to fertilize soil and produce substances relevant for human nutrition, such as amino acids, is one solution to reduce global CO_2_ emission tremendously. Due to the highly stable triple bond between the nitrogen atoms, it is difficult for microorganisms to fix N_2_ directly from the environment. Although this makes N_2_ unavailable as a biological nitrogen source for most organisms, some microorganisms from the domain of Archaea and Bacteria developed the trait to fix N_2_. This process is coupled to highly expensive metabolic costs of 16 ATP per N_2 _fixed^8–10^.

In Bacteria, diazotrophy is found in symbiotic and free-living species. The symbiosis ranges from the known example between legumes and intracellular diazotrophs, such as *Azobacter* spp. or *Pseudomonas* spp., to symbiotic relationships between diazotrophic bacteria and sponges, gymnosperms, or even insects^11^. Among the Archaea diazotrophy occurs among the methanogenic archaea (methanogens)^12–15^. Methanogens are anaerobic microorganisms and known for the ability to generate methane (CH_4_) as the end product of their energy metabolism. According to their substrate utilization spectrum, methanogens can be divided into different metabolic groups: hydrogenotrophic (H_2_, formate or simple alcohols), aceticlastic (acetate), methylotrophic (compounds containing a methyl group), H_2_-dependent methylotrophic (methylated compounds with H_2_ as electron donor), and methoxydotrophic (methoxylated aromatic compounds). Some methanogens are able to grow autotrophicly and hydrogenotrophicly by reducing CO_2_ with H_2_ to CH_4_, and play a crucial role in the global carbon cycle^16^. The first proof of diazotrophy in methanogens was shown in *Methanosarcina barkeri* strain 227 (DSM 1538)^17^ and *Methanothermococcus thermolithotrophicus* DSM 2095^18^. Since then, diatrozophy has been further studied in *M. thermolithotrophicus*^10,18^, *Methanococcus maripaludis*^19–21^, *M. barkeri*^22^, *Methanosarcina mazei*^23,24^, *Methanocaldococcus* sp.^14^ and in *Methanothermobacter marburgensis*^18^. One way to make use of diazotrophy in biotechnology could be to store the fixed N_2_ in nitrogen containing metabolic end products, such as proteinogenic amino acids.

Proteinogenic amino acids are applied in a variety of industrial sectors, such as food and feed, agriculture, pharmaceuticals, or packaging and housing. The production of amino acids through bacterial fermentation has marked an important branch of biotechnology for several decades^25^. Through genetic engineering of the main microorganisms for amino acid production, *Escherichia coli* and *Corynebacterium glutamicum*, were turned into highly optimized microbial cell factories for commercial production of amino acids^26–32^. However, the metabolic potential of archaea with regard to amino acid excretion has up to now been vastly overlooked^33–36^. In the emerging research and development field of Archaea Biotechnology^33,34^, the production of proteinogenic amino acids, which are important nutritional compounds, could be linked to the production of CH_4_, which is an important biofuel^35^. Several studies already showed the biotechnological potential of methanogens in biomethanation^37–47^. Moreover, methanogens were recently reported to excrete proteinogenic amino acids^35,48–51^. However, to our knowledge, the interplay between diazotrophy, amino acid production and CH_4_ production has not been described yet in the scientific literature.

The aim of our research was to examine the physiological and biotechnological characteristics of biological CO_2_- and N_2_-fixation in connection to proteinogenic amino acid excretion and CH_4_ production. Among five methanogens, *M. marburgenis* was prioritized to investigate N_2_-fixation, CH_4_ production, and amino acid excretion characteristics in closed batch cultivation mode at different NH_4_^+^ concentrations. Moreover, to unambiguously prove biological N_2_-fixation in a closed batch system and in a mass balance setting, a method for proving biological N_2_-fixation by methanogens without N-labelling techniques was also developed. The method focusses on headspace gas conversion in an isobaric setting in closed batch cultivation mode by undercutting a certain pressure – the theoretical threshold (THp_N2fix_). Since *M. marburgensis* was reported to be able to grow solely on N_2_^19^ and is able to excrete proteinogenic amino acids^35^ we hypothesized that *M. marburgensis* utilizes N_2_ as the sole source of nitrogen for proteinogenic amino acid production and excretion.

## Materials and methods

## Strains

The selection of hydrogenotrophic, autotrophic, diazotrophic methanogens was based on literature screening of the following articles^13,17–19,35,36,52^. From the analysis the following strains were selected for the experiments: *Methanothermobacter marburgensis* DSM 2133, *Methanobacterium thermaggregans* DSM 3266, *Methanocaldococcus villosus* DSM 22612, and *Methanothermococcus okinawensis* DSM 14208. All strains were taken from the strain collection of the Archaea Physiology & Biotechnology Group, Department of Functional and Evolutionary Ecology of the University of Vienna and had initially been obtained from the Deutsche Sammlung für Mikroorganismen und Zellkulturen GmbH (DSMZ) (Braunschweig, Germany). *Methanococcus maripaludis* S0001 was provided by Barny Whitman, University of Georgia, Athens, USA.

## Gases and chemicals

H_2_ (5.0), CO_2_ (5.0), N_2_ (5.0), H_2_/CO_2_ (20 Vol.-% CO_2_ in H_2_) (4:1), H_2_/CO_2_/N_2_ (11.13 Vol.-% N_2_ and 11.13 Vol.-% CO_2_ in H_2_) (7:1:1) were used for closed batch experiments. For gas chromatography (GC), N_2_/CO_2_ (20 Vol.-% CO_2_ in N_2_), CH_4_ (4.5) and the standard test gas (Messer GmbH, Wien, Austria) (containing 0.01 Vol.-% CH_4_, 0.08 Vol.-% CO_2_ in N_2_) were additionally used. All gases, except the standard test gas, were purchased from Air Liquide (Air Liquide GmbH, Schwechat, Austria).

## Media

*M. villosus* and *M. okinawensis* were grown in a chemically defined medium^53^. *M. thermaggregans* and *M. marburgensis *were cultivated in a minimal medium^37,54^ designated *M. marburgensis* medium (MM). *M. maripaludis* was grown in McN medium^40,55^.

Media were aliquoted into 117 mL serum bottles (VWR, Austria) to a total working volume of 50 mL and closed with blue rubber stoppers (20 mm, butyl rubber, CLS-3409-14, Chemglass Life Sciences, USA) and aluminum crimp caps (Ochs Laborbedarf, Bovenden, Germany). Before utilization, blue rubber stoppers were boiled 10 times for 30 min in Milli-Q water. To ensure anaerobic conditions the atmosphere in the headspaces of the closed serum bottles were changed before autoclavation by vacuuming and gassing with the respective gas mixture (H_2_/CO_2_ (4:1) or H_2_/CO_2_/N_2_ (7:1:1)) up to 2 bar rel. (3 bar abs.) repeating the procedure five times^53^. After autoclavation, sterile L-Cysteine-HCl·H_2_O, sterile NaHCO_3_ solution and/or sterile Na_2_S·9H_2_O were added to the sterile media in an anaerobic glove box (Coy Laboratory Products, Grass Lake, USA) prior to inoculation. For gassing, sterile syringe filters (w/0.2c µm cellulose, 514 − 0061, VWR International, USA) and sterile needles (disposal hypodermic needle, Gr 14, 0.60 × 30 mm, 23 G × 1 1/4′′, RX129.1, Braun, Germany) were used.

MM medium^40^ with varying NH_4_^+^ concentrations in regard to the usually provided NH_4_^+^concentration of 40 mmol L^− 138,54^ was prepared to examine N_2_-fixation characteristics of *M. marburgensis*. Moreover, the MM medium was prepared (as indicated) by omitting Na_2_CO_3_ to ensure that CO_2 _will be the sole source of carbon for growth and biomethanation^37^. To reach equal Na^+^ concentrations to the initial MM medium recipe, Na_2_CO_3_ was substituted by equal molarities of NaCl (**Supplementary Table ****1**). To replace L-cysteine monohydrate, a diluted HCl solution was used to retrieve the pH value. Medium without Na_2_CO_3_ was manually adjusted before gassing and autoclavation to pH 6.8 by titrating with 10 mol L^−1^ NaOH.

## Cultivations

Cultures were incubated in a water bath (GFL 1083, Burgwedel, Germany) at 65 °C (*M. marburgensis*,* M. thermaggregans* and *M. okinawensis*) or in a shaking air incubator at 37 °C (*M. maripaludis*) (GFL 3033, Burgwedel, Germany) and 80 °C (*M. villosus*) (ZWYR-2102 C, LABWIT Scientific Pty Ltd, Australia). For the purpose of N_2_-fixation all closed batch experiments were performed in a H_2_/N_2_/CO_2_ (7:1:1) gas mixture (unless otherwise indicated, for positive control). This study includes three different setups. The first setup is a screening experiment to prioritize the most suitable strain regarding N_2_-fixation. The second setup focuses to prove N_2_-fixation with destructive sampling technique. The third setup combines N_2_- and CO_2_-fixation and the excretion of amino acids.

To prioritize the most suitable strain, *M. marburgensis*, *M. maripaludis*, *M. thermaggregans*, *M. villosus* and *M. okinawensis* were grown in triplicates (*n* = 3) with one zero control to an OD_578_ of approximately 0.7. As the MM medium contains the highest NH_4_^+^ concentrations, it was additionally examined if growth of *M. marburgensis* was affected by reducing the amount of NH_4_^+^ to 10% of the original media NH_4_^+^ concentration. To remove residual nitrogenous compounds from the media, pre-cultured cells were washed (see below) before inoculation with reduced medium without NH_4_^+^. For a complete N-free media all cultures were washed three times, for all other experiments one time to none.

Experiments with only *M. marburgensis* were performed in quadruplicates (*n* = 4) or octuplicates (*n* = 8) at different NH_4_^+^ concentrations (0%, 1%, 5%, 7.5%, 10%, 25%, 50% and 100%) in relation to original media composition of 40 mmol L^−1^. A pre-culture with 10% of the initial NH_4_^+^ concentration of 40 mmol L^−1^ served as inoculum. To reduce the possibility of a NH_4_^+^ carryover some experiments as indicated were performed with one additional washing step. Washing of cells was performed by anaerobic transfer of 0.9 mL cell-culture into a 1.5 mL Eppendorf reactions tube (Eppendorf AG, Hamburg, Germany). The sample was centrifuged (5415 R, Eppendorf AG, Hamburg, Germany) at full speed (16,100 rcf) for 20 min. Supernatant was discarded and the pellet was resuspended in the respective medium. The closed batch experiment with 0% NH_4_^+^ served as negative control and gassed with H_2_/CO_2_ (4:1) as positive control. After every incubation time the serum bottles were left at room temperature for 45 min to cool down to reach isobaric conditions^53^. For the destructive sampling experiments, the cultures were not put back into the water bath for incubation.

For growth and N_2_-fixation experiments liquid samples of 0.75 mL were withdrawn from the serum bottles. Then growth was measured spectrophotometrically via OD (λ = 578 nm, blanked with Milli-Q water) (Beckman Coulter, DU 800 Spectrophotometer, California, USA). To sample and quantify amino acids, liquid samples of 1 mL were taken and centrifuged at full speed (16,100 rcf) for 30 min (5415 R, Eppendorf AG, Hamburg, Germany). The supernatant of each experiment was stored in a sterile Eppendorf tube at −20 °C until further analysis.

## Gas quantification

The relative pressure in bar within the serum bottle was measured with a digital manometer (LEO1-Ei, 1–3 bar rel., Keller GmbH, Winterthur, Switzerland). Gaseous substance (n / mol) in the serum bottles headspace was calculated via the ideal gas law. A non-inoculated serum bottle, gassed simultaneously with all other serum bottles served as a positive pressure control. As removing the needle releases some gas, pressure from this measurement indicated the “real pressure” and served as baseline for the calculations of N_2_-fixation. Headspace volume was determined in earlier experiments^53^ and adjusted after every OD measurement by the extracted sample volume of 0.75 mL. All measurements were performed at room temperature. Afterwards, the atmosphere was flushed and re-pressurized to 2 bar rel. (3 bar abs.). This procedure was repeated after every incubation or until the theoretical threshold (THp_N2fix_) was undercut. GC analyses were performed as previously described^56^. To obtain the actual amount of N_2_ the pressure inside the serum bottles was multiplied by 0.11392 based on the exact percentage of N_2_ (11.392 Vol.-%) in the gas mixture, then multiplied with the normalized gas composition gained from GC measurement. The values of the zero control served as N_2_ baseline. N_2_ uptake rate (NUR / mmol L^−1^ h^−1^) was calculated by dividing the deviation of N_2_ before and after incubation *(*ΔN_2_) by volume of the liquid medium and the time since last incubation (Δt).

7 $$\:NUR\left[\frac{mmol}{L\bullet\:h}\right]=\frac{{N}_{2}\left[mol\right]}{Vol.\left(med\right)\left[L\right]\bullet\:t\left[h\right]}$$

The specific nitrogen uptake (qN_2_ / mmol h^−1^ g^−1^) was determined by dividing the NUR by the biomass concentration (x / g L^−1^) calculated with an experimentally determined coefficient^53^.

8 $$\:q{N}_{2}\left[\frac{mmol}{h\bullet\:g}\right]=\frac{NUR}{x}$$

CO_2_ uptake rate (CUR / mmol L^−1^ h^−1^), H_2_ uptake rate (HUR / mmol L^−1^ h^−1^), CH_4_ evolution rate (MER / mmol L^−1^ h^−1^), carbon balance (C-balance), yields (Y_(CH4/CO2)_ and Y_(x/CO2)_) and biomass productivity (r_x_ / C-mmol L^−1^ h^−1^) was calculated as described elsewhere^53^.

## Theory

### Gas pressure and theoretical threshold (THp_N2fix_)

In a pure culture of a hydrogenotrophic, autotrophic methanogen full gas conversion must be considered^53^ for the optimal conditions of N_2_-fixation. The ideal gas composition for H_2_/CO_2_ (4:1) follows the stoichiometry (1):

1 $${\mathbf{4}}{\text{ }}{{\mathbf{H}}_{\mathbf{2}}}\,+\,{\mathbf{C}}{{\mathbf{O}}_{\mathbf{2}}} \to {\mathbf{C}}{{\mathbf{H}}_{\mathbf{4}}}\,+\,{\mathbf{2}}{\text{ }}{{\mathbf{H}}_{\mathbf{2}}}{\mathbf{O}}$$

The stoichiometry (2) of biological N_2_-fixation is:

2 $${{\mathbf{N}}_{\mathbf{2}}}\,+\,{\mathbf{8}}{\text{ }}{{\mathbf{H}}^+}+{\text{ }}{\mathbf{8}}{\text{ }}{{\mathbf{e}}^ - } \to {\mathbf{2}}{\text{ }}{\mathbf{N}}{{\mathbf{H}}_{\mathbf{3}}}\,+\,{{\mathbf{H}}_{\mathbf{2}}}$$

To catalyze this reaction and to break the highly energetic triple bond of N_2_ (945 kJ mol^−1^), 16 ATP and 8 electrons are required. One molecule of H_2_ and ammonia (NH_3_) is formed after the reduction of N_2_^9^. During artificial N_2_-fixation by the Haber-Bosch process, high pressure, high temperatures and a metal catalyst are necessary to form two molecules of NH_3_ out of three molecules of H_2_ and one molecule of N_2_. In theory, combining Eqs. (1) and (2) provides an ideal gas composition of seven parts of H_2_ and each one part of CO_2_ and N_2_ (3), with the possibility of full gas conversion by hydrogenotrophic, autotrophic, diazotrophic methanogens:

3 $${\mathbf{7}}{\text{ }}{{\mathbf{H}}_{\mathbf{2}}}\,+\,{\mathbf{C}}{{\mathbf{O}}_{\mathbf{2}}}\,+\,{{\mathbf{N}}_{\mathbf{2}}} \to {\mathbf{C}}{{\mathbf{H}}_{\mathbf{4}}}\,+\,{\mathbf{2}}{\text{ }}{{\mathbf{H}}_{\mathbf{2}}}{\mathbf{O}}\,+\,{\mathbf{2}}{\text{ }}{\mathbf{N}}{{\mathbf{H}}_{\mathbf{3}}}$$

All serum bottles were purged and re-pressurized after every incubation. A gassing manifold was used to ensure equal pressure of approximately 3 bar (abs.) of one closed batch (replicates and 1 zero control). To measure the exact total pressure after re-pressurizing, the THp_N2fix_ was calculated from the pressure of the zero controls. Under H_2_/CO_2_ gassing in a ratio of 4:1, the pressure can decrease to almost 0.6 bar abs., if all molecules of H_2_ and CO_2_ would be converted to CH_4_^53^:

**H**_**2**_**/CO**_**2**_**(4:1);** 3 bar (abs.)

4 $$\:p\left({\varvec{H}}_{2}\right)\frac{4}{5}\bullet\:3=\:2.4;\:p\:\left(\varvec{C}{\varvec{O}}_{2}\right)\frac{1}{5}\bullet\:3=\:0.6;\:p\:\left(\varvec{C}{\varvec{H}}_{4}\right)\:\frac{1}{5}\bullet\:3\:=\:0.6$$

Expecting full conversion, and neglecting biomass production, CH_4_ would be about one fifth of the pressure in the 7:1:1 gas mixture (see Eq. 3):

**H**_**2**_**/CO**_**2**_**/N**_**2**_**(7:1:1);** 3 bar (abs.)

5 $$\:p\left({\varvec{H}}_{2}\right)\frac{7}{9}\bullet\:3=\:2.33;\:p\left(\varvec{C}{\varvec{O}}_{2}\right)\frac{1}{9}\bullet\:3=0.33;\:p\left({\varvec{N}}_{2}\right)\:\frac{1}{9}\bullet\:3\:=\:0.33$$

If autotrophic, hydrogenotrophic methanogenesis takes place at full conversion, but without N_2_-fixation, one part of CH_4_, three parts of H_2_ and one part of N_2_ would remain in the **serum** bottle headspace:

6 $$\:[\frac{1}{9}\:{\text{C}\text{H}}_{4}\:+\:\:\frac{3}{9}{\text{H}}_{2}\:+\frac{1}{9}{\text{N}}_{2}]\:\bullet\:\:3\:=\frac{5}{3}\approx\:\:1.66$$

This leads to the theory, that a pressure below 1.66 bar (abs.) in this respective set-up is a clear indicator N_2_-fixation. To assure N_2_-fixation, gases was measured at the GC at a pressure of 1.5 (abs.).

### Ammonium determination

NH_4_^+ ^determination was performed using a modified procedure according to the method described before^57^. The oxidation solution, the colour reagent and the NH_4_Cl stock solution were prepared freshly before the measurement. As standards, nine different concentrations, ranging from 100 µmol L^−1^ to 1000 µmol L^−1^ of NH_4_Cl, were prepared. Samples were diluted with Milli-Q water to an end concentration between the standard ranges. Before the measurement, 300 µL of colour reagent and 120 µL oxidation solution were added immediately to the standards and samples and shortly mixed. After 30 min in the dark, measurement (λ = 660 nm) was performed using a 96 well plate (Microtest Plate 96 Well, F, Sarstedt AG & Co KG, Nümbrecht, Germany) with a plate photometer (Sunrise plate reader, Tecan Group AG, Männedorf, Switzerland). Regression curve R^2^ was always higher than 0.999.

### Amino acid analysis

For amino acid analyses the supernatant of samples was diluted with Milli-Q water at the ratio of 1:4. Measurements were performed on Agilent 1260 Infinity Bioinert HPLC system containing a fluorescence detector, a column oven, an autosampler and a quaternary pump. 1 mL of sample was mixed with 75 µL borate buffer (0.4 N in water, pH = 10.2; Agilent Technologies) followed by 5 µL OPA reagent, (3-mercaptopropionic acid in 0.4 mol L^−1^ borate buffer and 10 mg mL^−1^ of o-phthalaldehyde (OPA); Agilent Technologies). 100 µL of the mixture was injected to HPLC system after 2 min at 27 °C. Fluorescent derivates (primary dissolved free amino acids) were separated at 25 °C and a flow rate of 0.8 mL min^−1^ on a Zorbax ECLIPSE AAA column (4.6 × 150 mm, 3.5 μm particle size, Agilent Technologies) with a Zorbax ECLIPSE AAA guard cartridge (4.6 × 150 mm, 5 μm particle size, Agilent Technologies). The excitation wavelength was 340 nm and emission 450 nm. The use of a gain factors 9 or 10 was depended on the expected concentration and pre-tested before. For identification and quantification of peaks a primary amino acid standard mix (AAS18, Sigma Aldrich) in different concentrations was prepared for each run according to the concentration range of the samples (100 nmol L^−1^ to 15 µmol L^−1^). AAS18 standard mix lacks five amino acids (asparagine (Asn), glutamic acid (Glu), gamma-aminobutyric acid (GABA), taurine (Tau), tryptophan (Trp); Sigma Aldrich) which were added. In total, 20 different AA could be measured with this method. Valine and methionine had to be excluded from evaluation as they were located within a signal noise “ammonium peak” and therefore hard to measure within experiments with high NH_4_^+^concentrations as in our experiments. The details of this method were as previously described^36^.

## Results

### Prioritization of strains

**Fig. 1:**
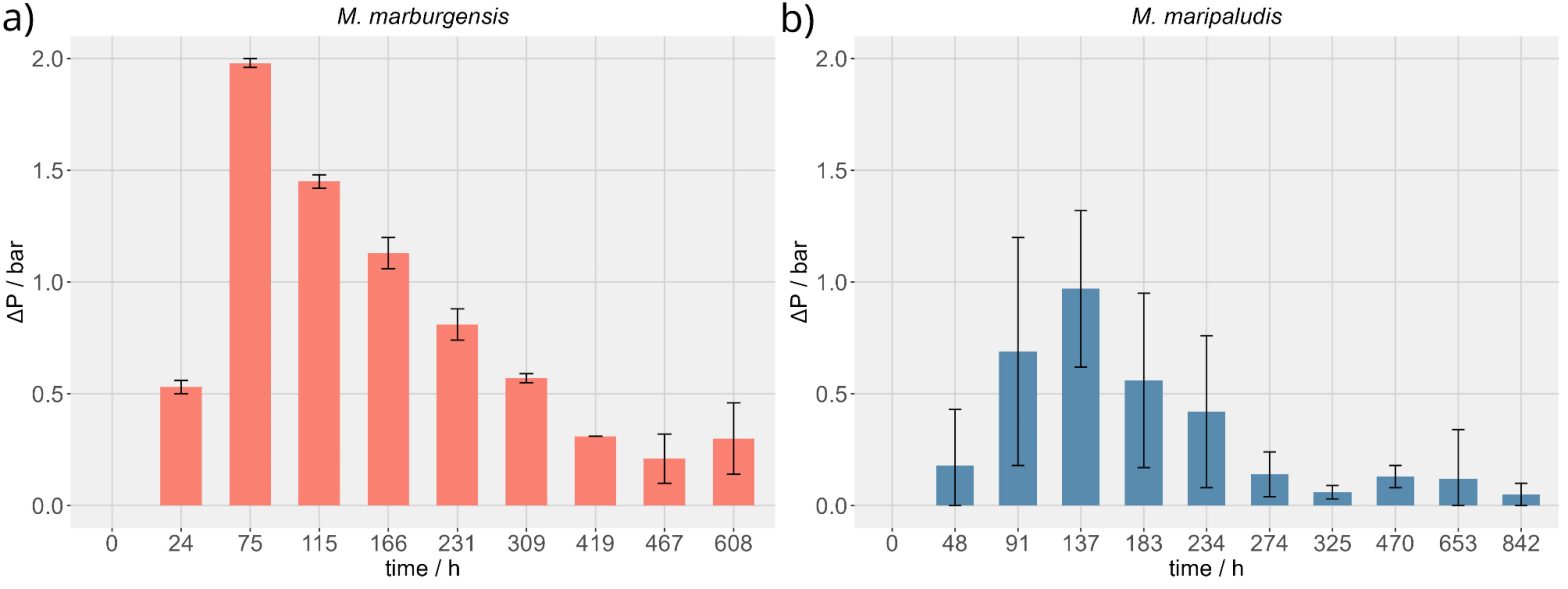
Time-series experiment showing the headspace pressure results of *M. marburgensis* (a) and *M. maripaludis* (b) in the prioritization phase. The ∆p of *M. marburgensis* reached up to 2 bar and outcompeted *M. maripaludis*. For all experiments *n* = 3.

Growth of *M. marburgensis*, *M. maripaludis*, *M. thermaggregans*, *M. villosus* and *M. okinawensis* was analyzed in NH_4_^+^ containing and an NH_4_^+^ free medium with an H_2_/CO_2_/N_2_ headspace. This allowed us to screen for NH_4_^+^ uptake, N_2_-fixation, amino acid excretion and the conversion of H_2_/CO_2_ to CH_4_ in parallel. All methanogens, excluding *M. thermaggregans*, could be grown to an OD_578_ of 0.7 in NH_4_^+^ containing medium (**Supplementary Fig. 1**). No growth or decrease in pressure (∆p) was detected when cells were washed and afterwards used as inoculum for another NH_4_^+^ free medium (data not shown). However, when *M. marburgensis* was grown in NH_4_^+^ free medium, without a biomass washing procedure the organism showed very slow growth (up to OD_578_ of 0.3 ) compared to optimal growth conditions^37^ while its ∆p reached up to 2 bar (Fig. 1a). *M. maripaludis* obtained a ∆p up to 1 bar (Fig. 1b). All other organisms showed neither growth nor a high and unambiguous ∆p (Fig. 1, **Supplementary Table 2**) when cultivated with NH_4_^+^ carryover. Therefore, only *M. marburgensis* was prioritized for further experiments. A subsequent transfer of *M. marburgensis* from NH_4_^+^ free medium, however, with NH_4_^+^ carryover, to another NH_4_^+^ free medium was not successful (data not shown). These experiments indicated that a certain amount of NH_4_^+^ was necessary for growth of *M. marburgensis* even with N_2_ in the headspace.

### N_2_-fixation ability of *M. marburgensis*

**Fig. 2:**
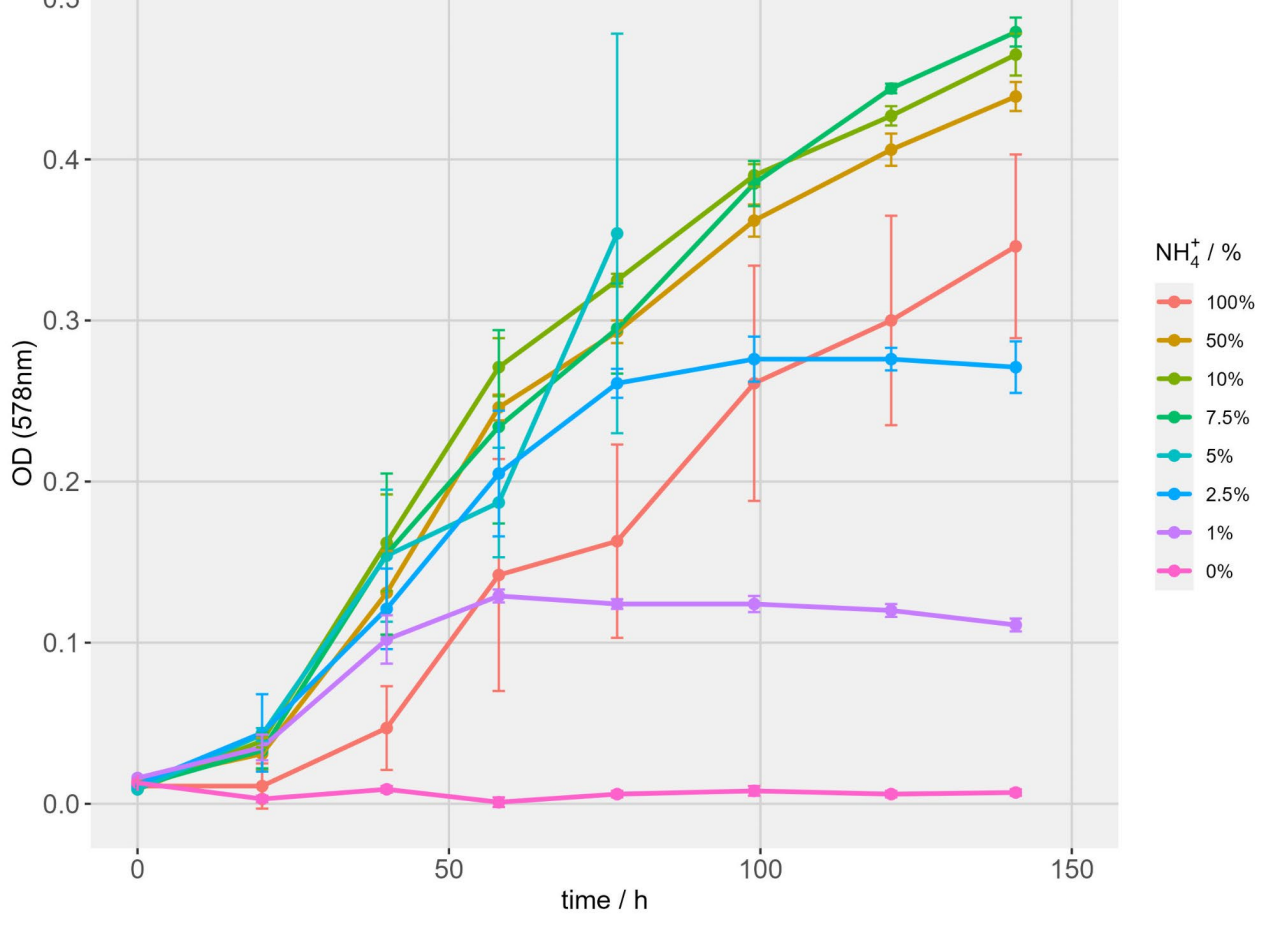
Growth kinetics of *M. marburgensis* during the THp_N2fix_ methodology experiment without Na_2_CO_3_ in the medium and at different NH_4_^+^ concentrations in relation to original media composition (*n* = 4). Due to undercutting the THp_N2fix_ in all four replicates of 5% NH_4_^+^ experiments at two subsequent time points, growth could not be monitored further since the method of destructive sampling has been employed for verifying THp_N2fix_ independently twice through GC measurements.

Growth and N_2_-fixation characteristics of *M. marburgensis* were monitored at different NH_4_^+^ concentrations in relation to original media composition of 40 mmol L^−1^ gassed with H_2_/CO_2_/N_2_. Therefore, pre-experiments were made to examine the effect on growth of NH_4_^+^ and carbonate (**Supplementary Fig. 2**,** Supplementary Fig. 3**). Due to chemical speciation reactions^58^, carbonate that is converted to CO_2_ serves as an additional carbon source for *M. marburgensis*. However, with an additional liquid-based carbon source our postulated theory to analyze THp_N2fix_ as an indicator for N_2_-fixation would be contradicted. In both set-ups (**Supplementary Fig. 2**,** Supplementary Fig. 3**) 0% and 1% NH_4_^+^ concentration showed no or very little growth with no N_2_-fixation. All other tested NH_4_^+^ concentrations followed a similar growth behavior and reached stationary phase after about 120 h of incubation. Experiments with carbonate added to the media resulted in faster growth. The THp_N2fix_ of 1.66 (abs.) was undercut several times during these experiments (Data Sheet 1: Pressure Drop CB, GC Sum). As the stationary phase of *M. marburgensis* was entered after about 120 h in the pre-experiments (**Supplementary Fig. 2**,** Supplementary Fig. 3**), growth and N_2_-fixation in the experiment was subsequently monitored until 141 h and additionally NH_4_^+^ concentrations of 2.5% and 7.5% have been included in the experiment (Fig. 2). End point measurements of NH_4_^+^ after 141 h showed that NH_4_^+^ limitation only occurred in 0% and in 5% NH_4_^+^ experiments (data not shown).

**Fig. 3:**
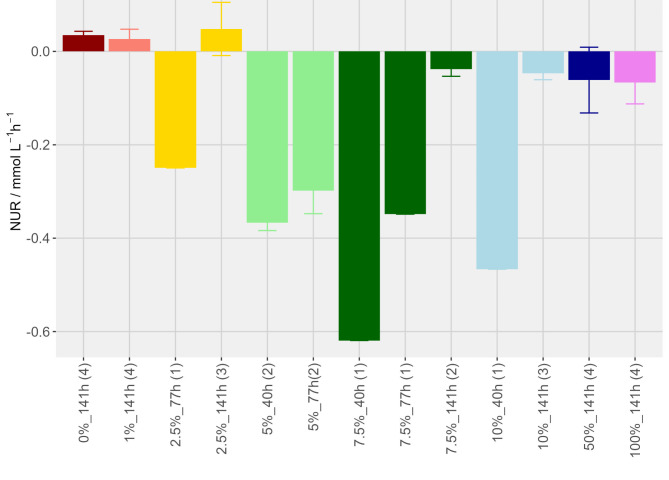
NUR / mmol L^−1^ h^−1^ of *M. marburgensis* with different NH_4_^+^ concentrations calculated by the THp_N2fix_ methodology. Negative values for NUR / mmol L^−1^ h^−1^ on the y-axis indicate N_2_-fixation. Sample size varies due to the time point of undercutting THp_N2fix_. The sample number (n) is indicated in brackets on the x-axis. In addition, the sampling time points (in h) and the values of the applied NH_4_^+^ concentration (in %) are also indicated on the x-axis.

THp_N2fix_ was undercut in several samples at different time points between 40 and 77 h of incubation and the headspace gas composition has been analyzed via GC for determinations of NUR (Fig. 3). Highest NUR of 0.62 mmol L^−1^ h^−1^ after 40 h of incubation was obtained when 7.5% NH_4_^+^ concentration had been examined. Only at 0% and 1% NH_4_^+^ the pressure never undercut THp_N2fix_. The quantification of NUR through the theoretical background of the THp_N2fix_ methodology could thus be verified (Fig. 3). It became visible that after the first time of undercutting THp_N2fix_ and with every further incubation round, THp_N2fix_ could hardly be reached anymore and the physiological ability for N_2_-fixation seemed to be reduced as NUR is decreased. Moreover, in addition to the THp_N2fix_ methodology, N_2_-fixation of one replicate of the 10% NH_4_^+^, two replicates of the 7.5% NH_4_^+^, all replicates of the 5% NH_4_^+^ and one replicate of the 2.5% NH_4_^+^ cultures were analyzed via GC. Therefore, the destructive sampling method was used in order to independently prove N_2_-fixation of *M. marburgensis* by GC in addition to the already available proof of N_2_-fixation through the THp_N2fix_ methodology (Fig. 3, Data Sheet 1: Sum NUR). The culture that was cultivated at 100% NH_4_^+^ did surprisingly not grow as expected from previous experiments (Fig. 2) and reached THp_N2fix_ at a low mean value at the last measurement time-point (Fig. 3). The qN_2_ of *M. marburgensis* that was reached in both replicates of 7.5% NH_4_^+^ was 10.24 mmol h^−1^ g^−1^ at 40 h and 9.99 mmol h^−1^ g^−1^ at 77 h (Data Sheet 2: NUR_qN2). HUR, CUR, NUR, MER and the mass balances were calculated from GC measurements (**Supplementary Table 3**). NUR was always higher at earlier time points when THp_N2fix_ was undercut for the first time. Full conversion of H_2_ and CO_2_ to CH_4_ did not occur in 0% and 1% replicates and therefore did not drop below THp_N2fix_. The ratios of HUR:CUR, calculated from HUR/CUR and C-balances under consideration of biomass production from the total of Y_(CH4/CO2)_ and Y_(x/CO2)_, were found to be partially inconsistent in measurements where NUR was higher.

### Uptake of NH_4_^+^ and N_2_ for amino acid excretion

**Fig. 4:**
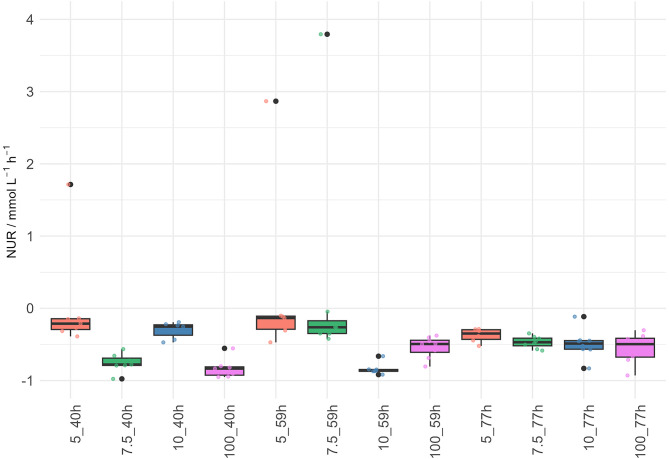
Results of NURs during the amino acid excretion and N_2_-fixation time series experiment. The experiment was performed using destructive sampling procedure. *M. marburgensis* biomass was washed before inoculation. NUR / mmol L^-1^h^-1^ of *M. marburgensis* during growth on H_2_/CO_2_/N_2_ are shown as individual box plots indicating NH_4_^+^ concentration and the time point. The first number on the x-axis labelling indicates the percentages of NH_4_^+^ in the medium. The first number on the x-axis labelling indicated the sampling time point. The legend on the right-hand side of the graph indicates the respective group in colours (red: 5% NH_4_^+^, green: 7.5% NH_4_^+^, blue: 10% NH_4_^+^, magenta: 100% NH_4_^+^ as well as the time point of sampling (40 h, 59 h, 77 h). All results are *n* = 8. Thus, 93 out of 96 individual NUR results show N_2_-fixation whereas only 3 NUR results from 3 different set-ups do not show N_2_-fixation.

For visualization of N_2_-fixation and the simultaneous production of proteinogenic amino acids, experiments of *M. marburgensis* were performed with 0%, 5%, 7.5%, 10% and 100% NH_4_^+^, gassed with H_2_/CO_2_/N_2_. To reduce the possibility of a NH_4_^+^ carryover the experiments were performed with one washing step before inoculation in octuplicates (*n* = 8) and using a second zero (gas) control. Due to this additional biomass washing step slower growth compared to non-washed biomass experiments was observed (please compare growth curves shown in Fig. 2, **Supplementary Fig. 2**, **Supplementary Fig. 3** to **Supplementary Fig. 4**). After 77.17 h of incubation the OD_578_ reached values between 0.17 and 0.20. As expected, the 100% NH_4_^+^ H_2_/CO_2_ positive control showed the highest OD_578_ value of around 0.25. Gas samples were taken after approximately 40, 59 and 77 h of incubation (**Supplementary Fig. 4**). An NH_4_^+^ uptake during N_2_-fixation is evident in all experiments (**Supplementary Fig. 5**), although NH_4_^+^ was never completely consumed. The highest uptake was achieved in the positive control (Data Sheet 1: NH4Cl Uptake). Highest NUR was calculated from 7.5% to 100% closed batch experiments after 40 h of incubation with 0.91 or 0.83 mmol L^−1^ h^−1^, respectively and from 10% after 59 h with 0.88 mmol L^−1^ h^−1^. The qN_2_ follows the same pattern (**Supplementary Table 3**). As a general trend, earlier sampling time points rather show higher NUR values, and later time points lower but more balanced NUR values. (Fig. 4). A detailed description of HUR, CUR, and MER is shown in **Supplementary Table 3**. MER during N_2_-fixing conditions and omission of carbon in the media could not reach the MER values previously described for closed batch experiments^37,40,53^.

**Fig. 5:**
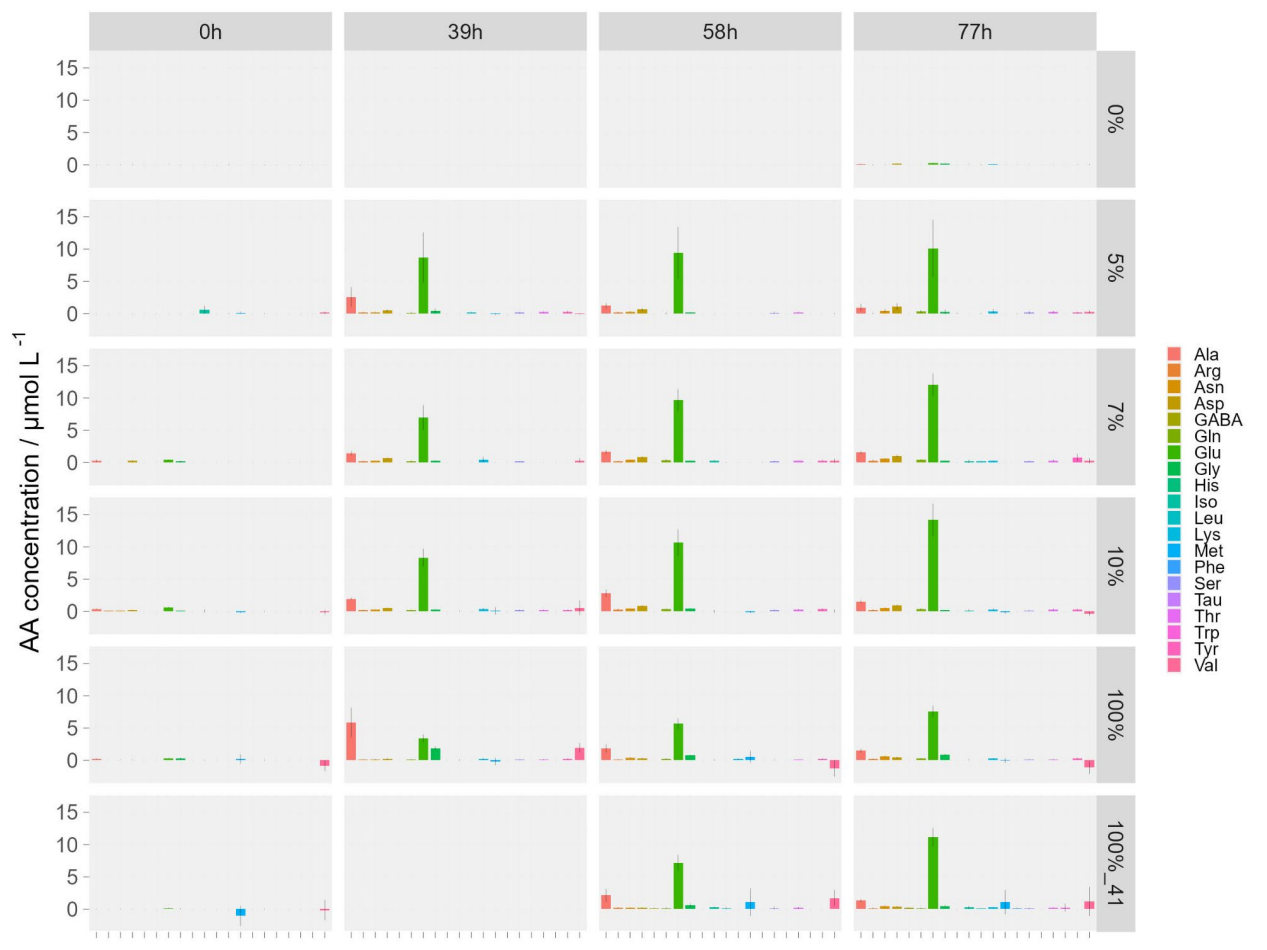
Results of individual amino acid concentrations during the amino acid excretion and N_2_-fixation time series experiment. *M. marburgensis* biomass was washed before inoculation. Amino acid concentration / µmol L^−1^ of *M. marburgensis* during N_2_-fixation on H_2_/CO_2_/N_2_ shown as individual bar charts with standard deviation for each amino acid. The legend on the right-hand side of the graphs indicates the quantified amino acids. On the left-hand y-axis, the amino acid (AA) concentration / µmol L^−1^ is shown. NH_4_^+^ concentrations of the respective time series are indicated on the right-hand y-axis from top to bottom: 0%, 5%, 7.5%, 10%, 100% and 100%_41. 100%_41 denotes amino acid excretion during incubation with H_2_/CO_2_ (4:1). The sampling time is shown on the x-axis as headers from left to right. After biomass washing negligible amino acid concentration is measurable at time point 0 h. All results are *n* = 8.

Independent of the NH_4_^+^ concentration almost all detectable proteinogenic amino acids were found in the supernatant (Fig. 5). The highest excreted amino acids are glutamic acid (Glu), alanine (Ala), glycine (Gly) and asparagine (Asn). The concentrations of Glu, Gly and Asn are constantly increasing during the time-course of the cultivation, whereas the trend for Ala is not entirely clear. It could be that Ala is partially consumed during the cultivation. All amino acid excretion experiments showed a clear NH_4_^+^ dependence, where amino acid concentrations at 5%, 7.5% and 10% of NH_4_^+^ is different to 100% NH_4_^+^ (in H_2_/CO_2_ and H_2_/CO_2_/N_2_) (Fig. 5). The highest amino acid excretions rates were detected for Glu with up to 4.59 µmol L^−1^ h^−1^ in 5% after 40 h and a smaller amount of Ala with up to 1.36 µmol L^−1^ h^−1^. Highest value of Gly was achieved in 100% after 40 h with 0.99 µmol L^−1^ h^−1^ (Data Sheet 1: HPLC Productivity).

Examining the total amount of excreted amino acids, an increasing excretion of amino acids over time with the highest concentration of 14.67 to 18.44 µmol L^−1^ in later time points was found (Data Sheet 1: AUR vs. AA). The presence of higher concentrations of NH_4_^+^in the media seems to serve as an inhibitor for amino acid production, which is in agreement to earlier findings^36^, as 100% experiments showed slightly lower excreted amino acid concentrations (Fig. 5, Data Sheet 1: HPLC Productivity). A comparison of the total uptake of NH_4_^+^ with the total amino acids excretion rate indicated that amino acid excretion rate increased with increasing AUR. Furthermore, during depletion of NH_4_^+^, the concentration of AA did not increase (**Supplementary Fig. 5**).

## Discussion

Agriculture and the production of artificial N-fertilizers are an indirect source of GHG emissions through releasing N_2_O via nitrification of ammonia (NH_3_)^2,4^. To generate this ammonia fertilizer, the Haber-Bosch process is the main industrial procedure for synthetic N_2_-fixation and responsible for a release of 1.5 tons of CO_2_ per ton of NH_3_ produced^6^. Therefore, the identification of microbial strains optimized for molecular N_2_-fixation could reduce the amount of chemically produced ammonia via the Haber-Bosch process. Physiologically, an ideal strain for N_2_-fixation in a biorefinery concept should include certain properties, such as high specific growth rate, high specific CO_2_ conversion and high N_2_-fixing abilities. Furthermore, the strain should be cultivatable in chemically defined minimal medium exclusively with inorganic compounds and substrates. Among the methanogens, several candidates would fulfill these prerequisites. For this reason, suitable methanogens should be identified using a fast and simple screening methodology regarding their N_2_-fixation characteristics. Therefore, this study proposes a fast and easy detection method of diazotrophic characteristics of autotrophic, hydrogenotrophic methanogens using THp_N2fix_ as proof of N_2_-fixation and concomitant amino acid production. Moreover, in the context of “power to gas” technology, biomethanation of CO_2_^43–45,48^ in combination with N_2_-fixation for the excretion of amino acids could become of high economic interest^48–51^. To our knowledge there is no study yet that examined combined CO_2_- and N_2_-fixation regarding amino acid excretion. However, this study should only be seen as proof of principle. For a high throughput screening of THp_N2fix_ and quantification of NUR, online measurements of the headspace pressure would be clearly desirable^40,41^. In addition, in-line or at-line measurements of biomass and of NH_4_^+^ concentrations would be needed to be able to examine the growth and production kinetics of methanogens under N_2_-fixing conditions.

Furthermore, methanogens may use carbonates as an additional carbon source^59^. A carbon source in the media would render the THp_N2fix_ method as an indicator for N_2_-fixation impossible, as additional H_2_ utilization beyond the 4:1 ratio would potentially occur. For that reason, it was necessary to exclude carbonate from the media for the purpose of converting CO_2_ to CH_4_ only from the gas that had been supplied in the serum bottle headspace. Gassing with H_2_/CO_2_/N_2_ generally resulted in lower growth (**Supplementary Fig. 4**) when compared to gassing with only H_2_/CO_2_ (**Supplementary Fig. 2**,** Supplementary Fig. 3**). This could be explained via constrained growth by the expensive N_2_-fixing process^9^. Within this set-up, undercutting the THp_N2fix_ while fixing N_2_ was successful in experiments where NH_4_^+^ was limiting, ranging between 2 and 10% and in a time frame from 40 to 77 h of cultivation (Fig. 3). The qN_2_ of *M. marburgensis* (Data Sheet 2: NUR_qN2) detected in this study are in the range 5–20% the maximum specific CH_4 _production rates^54^. This high physiological capacity of *M. marburgensis* for diazotrophic growth seems to agree with that of *M. thermolithotrophicus*^10^.

Regarding the capability of methanogens for N_2_-fixation, *M. marburgensis* harbours a *nif* gene cluster^13^ and the organism was already examined in continuous culture regarding its growth yield under N_2_-fixing conditions^18^. Thus, *M. marburgensis* has been shown to be capable to growing on NH_4_^+^-free medium solely using N_2_ as nitrogen source. However, here we show that NH_4_^+^ addition is required to enable N_2_-fixation of *M. marburgensis* and that there seems to be an optimal concentration of NH_4_^+^ between 2.5 and 10% for optimal N_2_-fixation. At higher concentrations of NH_4_^+^ (50% and 100%) (Fig. 3), the pressure rarely dropped below the THp_N2fix_, and it seemed that N_2_-fixation was slightly inhibited or at least reduced. This could be related to the fact that *M. marburgensis* is usually grown at NH_4_^+^ concentrations of 20 to 40 mmol L^− 138,40,53,54^ which acts as the preferred nitrogen source, or that the tested NH_4_^+^ concentrations above 20 mmol L^−1^ physiologically down-regulates the nitrogenase activity^20,60^. NH_4_^+^ switch off can occur in methanogens. This means that there is an inactivation of N_2_-fixation once a thermodynamically superior nitrogen source is available^20^. A switch-off of the nitrogenase activity with addition of NH_4_^+^ as a superior and more easily accessible nitrogen source, as seen in *M. maripaludis*^60^ could not be fully confirmed here. However, during growth of *M. maripaludis *on Ala only a partial switch-off of the nitrogenase activity has been observed^60^. Thus, it might be possible that in *M. marburgensis* the uptake of Ala allows for N_2_-fixation in the presence of NH_4_^+^ (**Supplementary Fig. 5**). N_2_ fixation in the presence of NH_4_^+^ was studied e.g., in *M. barkeri*^17^ and in *M. maripaludis*^60^, but in both studies N_2_-fixation in the presence of NH_4_^+^ had not been observed. Except for our study, N_2_-fixation in the presence of NH_4_^+ ^has only been reported in a single experiment^61^. In experiments with washed *M. marburgensis* biomass compared to non-washed biomass, metabolic expensive N_2_-fixation was proven even with higher NH_4_^+^ concentrations (Figs. 3 and 4). This might have occurred due to the slow growth and introduced stress because of the washing step. Thus, amino acid excretion could also be the reason for the partially inconsistent and higher HUR:CUR ratio and C-balances (**Supplementary Table 3**).

The results presented in this study confirm that *M. marburgensis* is a diazotrophic organism. However, we could not yet confirm earlier findings that the sole N-source of *M. marburgenis* can be N_2_^19^. In independent unpublished experiments with *M. marburgensis* using N_2_ as sole source of nitrogen, the organism did also not grow or produce CH_4_ (Nevena Maslać, personal communication). We show that the N_2_ is converted by *M. marburgensis* during growth on H_2_/CO_2_/N_2_ in chemically defined minimal medium into proteinogenic amino acids (Fig. 5) which are excreted into the growth medium. One could argue that the amino acids were not actively or passively excreted. However, in a previous study it has been shown that cell lysis was not a substantial source of amino acid excretion by *M. marburgensis*^35^. In this study a variety of amino acids were excreted by *M. marburgensis* with the highest total amount of up to 7.5 µmol L^−1^ h^−1^ in early time points (Data sheet 1: HPLC Productivity). Interestingly, Ala seems to be consumed in later time points of 5%, 7.5% and 10% experiments (Fig. 5). This finding might provide insight for the varying NUR (Fig. 4), as NH_4_^+^ could hinder nitrogenase enzyme activity, whereas the utilization of Ala could switch to an intermediate regulatory response^60^.

Amino acid excretion rates of *M. marburgensis* under N_2_-fixing conditions can currently not compete with the genetically engineered and high amino acid producing organisms *C. glutamicum* and *E. coli*^26^. Highest concentrations of Glu reached 0.045 g L^−1^ compared to genetically modified *C. glutamicum* of 40 g L^−1^ (Table 1). Nevertheless, they succeeded to create a modified *C. glutamicum* from a wild type with no L-Lysine production to 0.6 to 4.0 g L^−1^ h^−128^. Furthermore, conventional amino acid excreting cell factories are engineered to only overproduce one specific amino acid and are, unlike *M. marburgensis*, not able to fix atmospheric N_2_ for their production^26,29^. Moreover, the carbon and energy substrate for *E. coli* and *C. glutamicum* are carbohydrates and *M. marburgensis* utilizes CO_2_ and H_2_. With the synthetic biology tools that have become available to genetically enhance methanogens^55,62–66^, some of these organisms may become cell factories for proteinogenic amino acid production.

**Table 1:** Comparison of amino acid concentrations from this study with *M. marburgensis* to *E. coli* and *C. glutamicum*.

Organism	Amino acid	Closed batch value this study / g L^−1^	Value from other studies / g L^−1^	Reference
*C. glutamicum* ^1^	Asp	0.0031	0.46	^67^
*C. glutamicum* ^1^	Leu	n.d.	0	^68^
*C. glutamicum* ^1^	Lys	0.0014	0	^27^
*C. glutamicum* ^2^	Ala	0.0109	110	^26^
*C. glutamicum* ^2^	Arg	0.0011	92	^69^
*C. glutamicum* ^2^	Gln	0.0015	73.5	^70^
*C. glutamicum* ^2^	Glu	0.0448	40	^26^
*C. glutamicum* ^2^	Ile	n.d.	32	^71^
*C. glutamicum* ^2^	Leu	n.d.	20	^68^
*C. glutamicum* ^2^	Lys	0.0014	120	^27^
*C. glutamicum* ^2^	Met	n.d.	7	^72^
*C. glutamicum* ^2^	Ser	0.0005	44	^73^
*C. glutamicum* ^2^	Try	0.0030	65	^26^
*E. coli* ^2^	Ala	0.0109	114	^74^
*E. coli* ^2^	Phe	0.0001	60	^26^
*E. coli* ^2^	Thr	0.0008	90	^26^
*E. coli* ^2^	Val	n.d.	60–70	^26^

n.d.: not detected.

^1^wild-type microorganism.

^2^genetically engineered microorganisms.

## Conclusions

This study sheds new light on the link between methanogenesis, biological N_2_-fixation, and proteinogenic amino acid excretion. We show the inherent industrial potential of *M. marburgensis* for amino acid excretion under N_2_-fixing conditions. The THp_N2fix_ method can be used to determine N_2_-fixation rates of hydrogenotrophic, autotrophic, diazotrophic methanogens – without the need for GC measurements. Moreover, the THp_N2fix_ methodology may serve as the basis for establishing high-throughput screening of methanogens and other gas-fermenting organisms where a pressure drop or increase occurs. This study has implications for research in microbial physiology, ecology, and biotechnology of amino acid excretion, methanogenesis and N_2_-fixation and may serve as basis for developing applications in gas fermentation and Archaea Biotechnology.

## Electronic supplementary material

Below is the link to the electronic supplementary material.

Supplementary Material 1

## Data Availability

The datasets generated and/or analysed during the current study are available in the PHAIDRA repository of Universität Wien, https://doi.org/10.25365/phaidra.515; https://doi.org/10.25365/phaidra.516.
