# Supplementary material for: Quantitative analysis of amino acid excretion by Methanothermobacter marburgensis under N2-fixing conditions
Source: Sci Rep. 2025 Jan 30;15:3755. doi: 10.1038/s41598-025-87686-1 (PMC11782530; doi:10.1038/s41598-025-87686-1)
Supplement: Supplementary file 1 — Supplementary Material 1 [file 41598_2025_87686_MOESM1_ESM.docx]

**Supplementary material to: Quantitative analysis of amino acid excretion by *Methanothermobacter marburgensis* under N_2_-fixing conditions**

Barbara Reischl^1,2^, Benjamin Schupp^1^, Hayk Palabikyan^1^, Barbara Steger-Mähnert^3^, Christian Fink^2^,

Simon K.-M. R. Rittmann^1,2,*^

^1^Archaea Physiology & Biotechnology Group, Department of Functional and Evolutionary Ecology, Universität Wien, Wien, Austria

^2^Arkeon GmbH, Tulln a.d. Donau, Austria

^3^Marine Biology/Microbial Oceanography, Department of Functional and Evolutionary Ecology, Universität Wien, Wien, Austria

*Corresponding author:

Dr. Simon K.-M. R. Rittmann, Privatdoz.

Archaea Physiology & Biotechnology Group

Department of Functional and Evolutionary Ecology

Universität Wien

Djerassiplatz 1

1030 Wien

Austria

Tel.: +43-4277-76513

eFax.: +43-4277-876513

Email: [simon.rittmann@univie.ac.at](mailto:simon.rittmann@univie.ac.at)

**Supplementary figures**


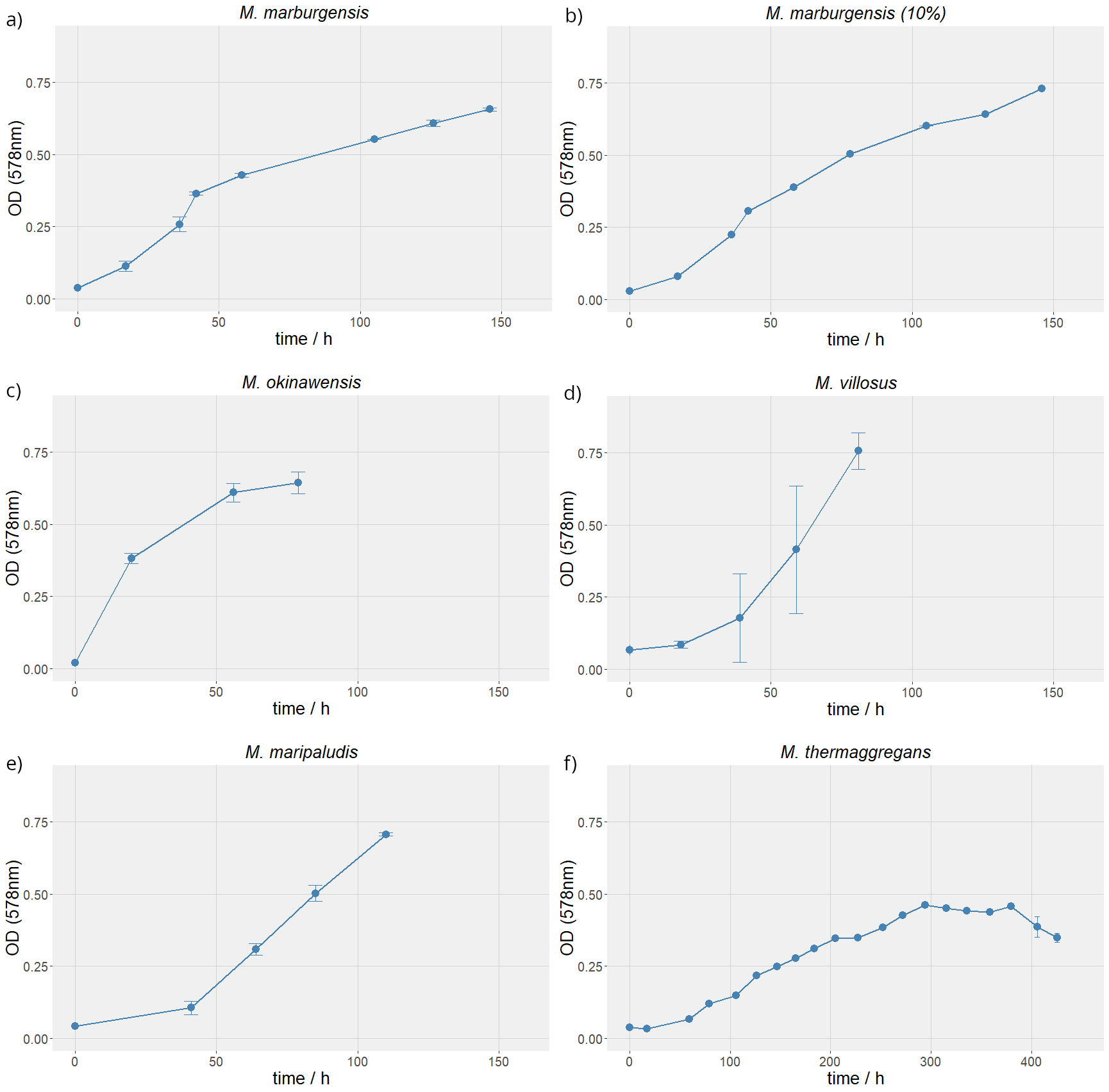
**Supplementary figure 1**: Growth kinetics of methanogens for prioritization. *M. marburgensis* (a), *M. marburgensis* with 10% of NH_4_^+^ in the media (b), *M. okinawensis* (c), *M. villosus* (d), *M. maripaludis* (e) and *M. thermaggregans* (f).


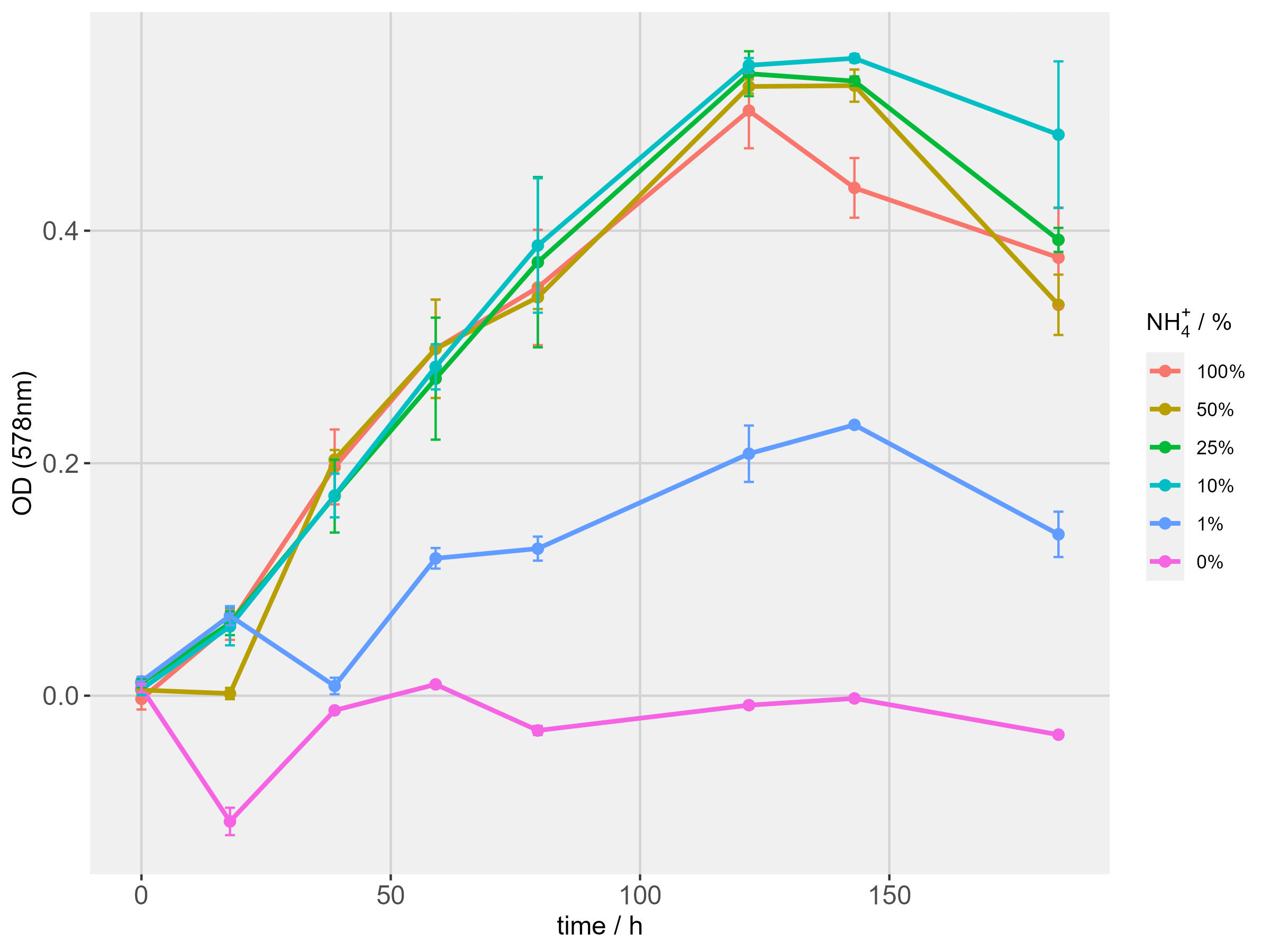
**Supplementary figure 2**: Growth curve of pre-experiments of *M. marburgensis* (n = 4) with different concentration of NH_4_^+^ in the medium.


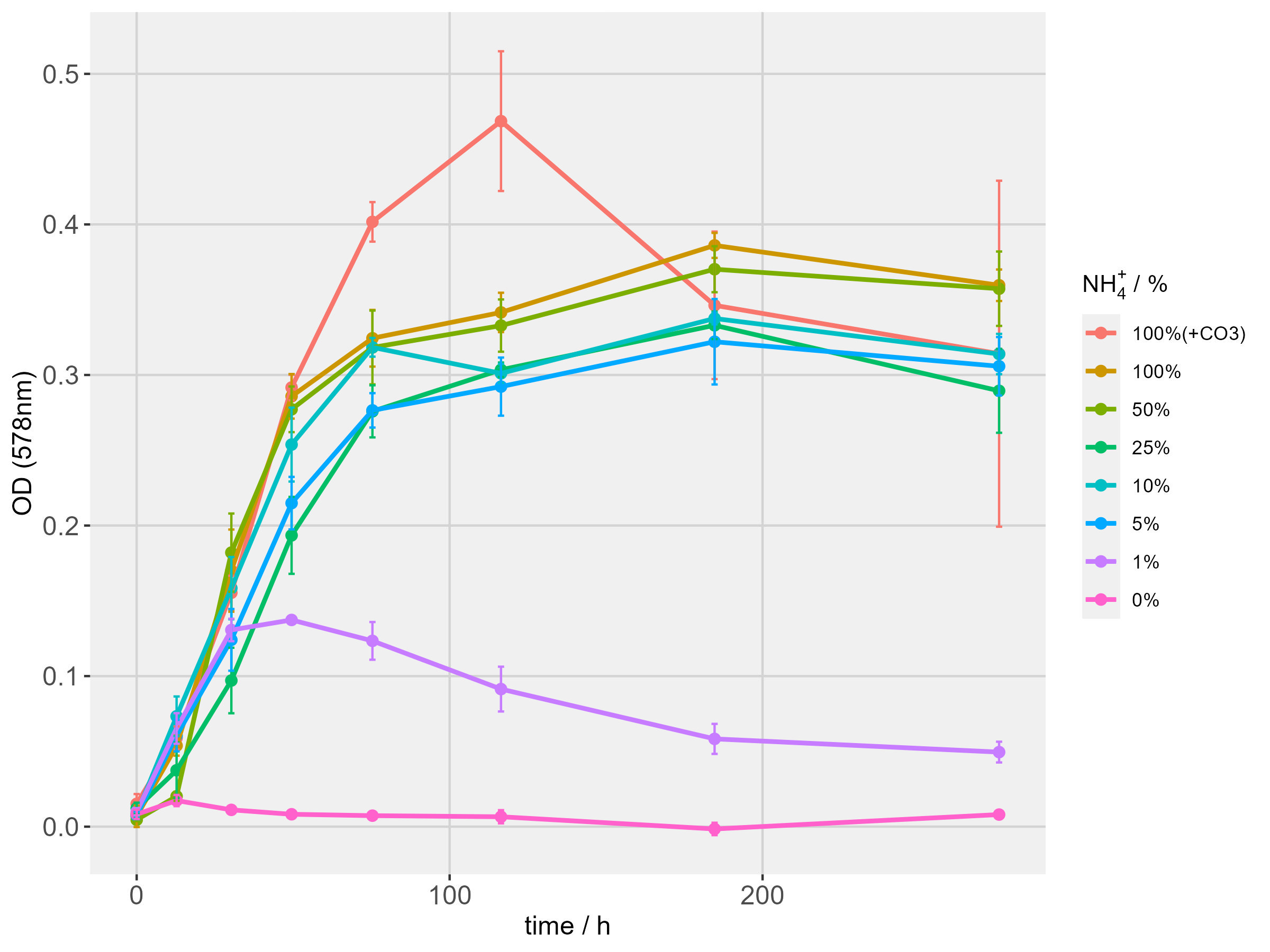
**Supplementary figure 3**: Growth curve of pre-experiments of *M. marburgensis* (n = 4) with different concentration of NH_4_^+^ in the medium. More concentrations are included as well as carbonate in the media.


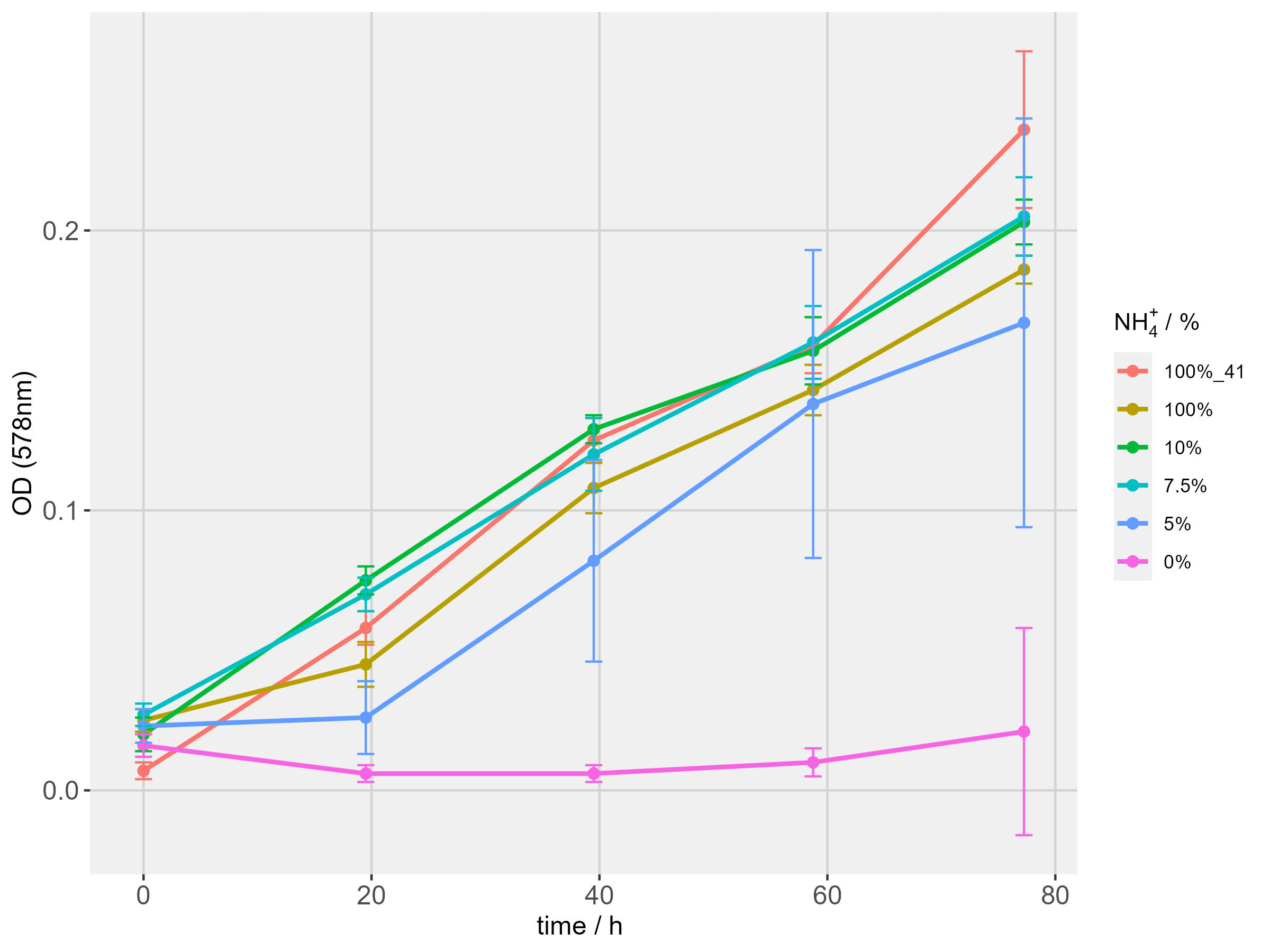
**Supplementary figure 4**: Growth kinetics OD_578nm_ of *M. marburgensis* (n = 8) with varying NH_4_^+^ concentrations in relation to original medium gassed with H_2_/N_2_/CO_2_ (7:1:1). Gassing with H_2_/CO_2_ (4:1) (100%_41) served as positive control. Experiments were performed with one washing step and gas samples taken after 40, 59 and 77 h. Samples after re-gassing were returned into the water bath for further incubation.


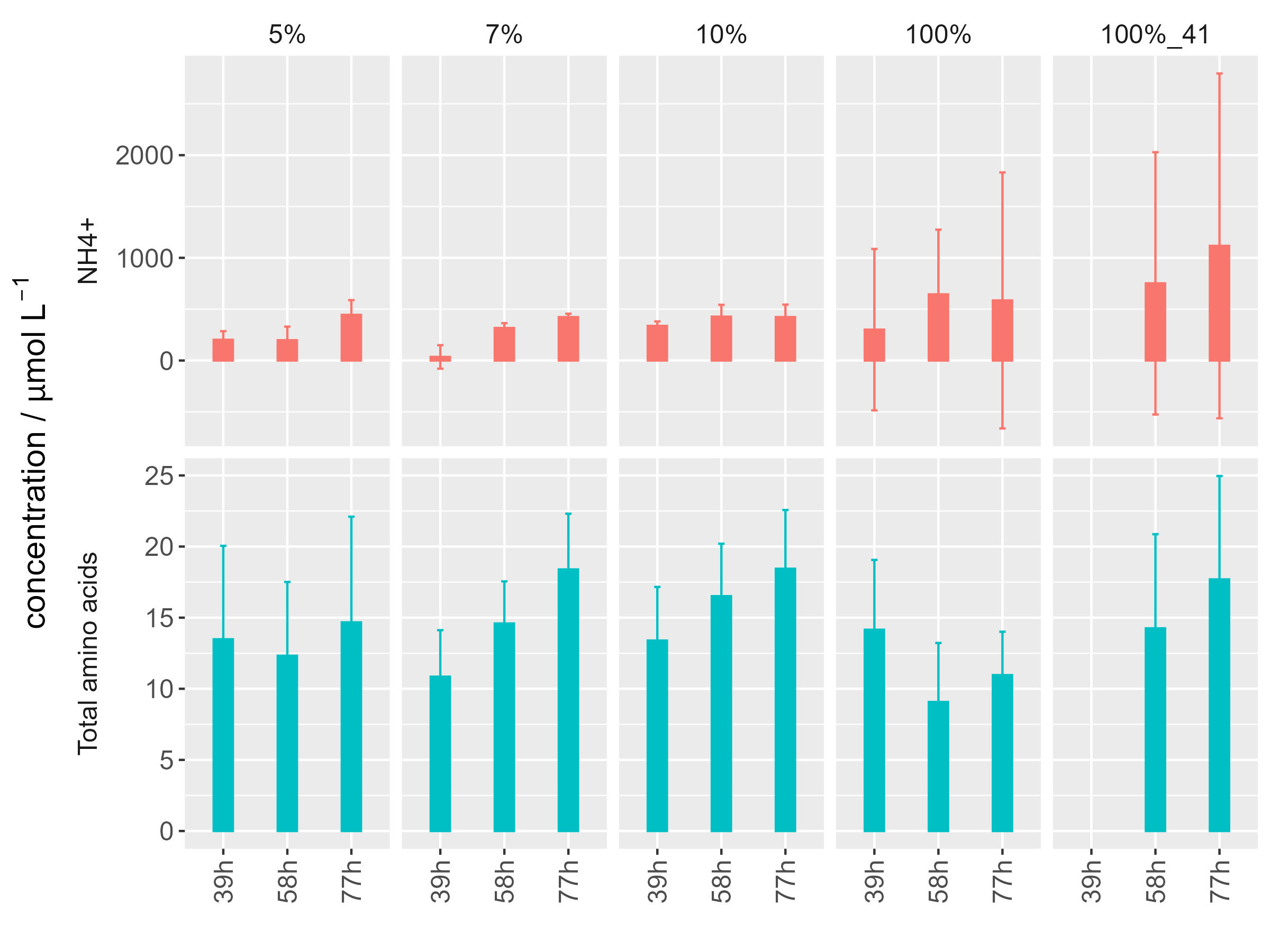
**Supplementary figure 5**: Concentrations of ammonium chloride (NH_4_^+^ / µmol L^-1^) are shown as positive values in the upper panels in salmon. Concentrations of the total amount of excreted amino acids (Total amino acids / µmol L^-1^) are shown in the lower panels in turquoise. The amount of excreted amino acids is increasing with increasing uptake of NH_4_^+^, which can be unambiguously seen in the experiments with 7% and 10% ammonium chloride.

**Supplementary tables**

**Supplementary table 1**: Composition of MM medium with varying NH_4_^+^ concentrations in relation to original MM medium in g L^-1^. Percentage of NH_4_Cl in relation to original media. -/+ presents the absence and presence of Na^2^CO_3_.

| Compound / g L^-1^ | 100% + | 100% - | 50% - | 25% - | 10% - | 5% - | 1% - | 0% - |
| --- | --- | --- | --- | --- | --- | --- | --- | --- |
| KH_2_PO_4_ | 6.8 | 6.8 | 6.8 | 6.8 | 6.8 | 6.8 | 6.8 | 6.8 |
| Na_2_CO_3_ | 3.6 | 0 | 0 | 0 | 0 | 0 | 0 | 0 |
| NH_4_Cl | 2.1 | 2.1 | 1.05 | 0.525 | 0.21 | 0.105 | 0.021 | 0 |
| NaCl | 0 | 2 | 2.15 | 2.725 | 3.07 | 3.185 | 3.277 | 3.3 |
| mL L^-1^ |  |  |  |  |  |  |  |  |
| TE 200x | 5 | 5 | 5 | 5 | 5 | 5 | 5 | 5 |

**Supplementary table 2**: Decrease of pressure (∆p) and final OD_578_ of methanogens in N-free medium

| **Strain** | **Pressure (Δp)**  **/ bar** | **Growth_max_**  **(OD_578_)** | **time**  **/ h** |
| --- | --- | --- | --- |
| *M. marburgensis* | 2 bar | 0.30 | > 600 |
| *M. maripaludis* | 1 bar | 0.04 | > 980 |
| *M. villosus* | no | no | > 920 |
| *M. okinawensis* | no | no | > 920 |

**Supplementary table 3**: HUR, CUR, NUR, MER, ratios, r_(x)_, product yields, and C-balance of *M. marburgensis*^+^

| **Sample** | **HUR /  mmol L^-1^ h^-1^** | **CUR /  mmol L^-1^ h^-1^** | **NUR /  mmol L^-1^ h^-1^** | **MER /  mmol L^-1^ h^-1^** | **Ratio 4:1 HUR:CUR** | **Ratio 1:1  CUR:MER** | **r_(x)_ /  c-mmol L^-1^ h^-1^** | **Y_(CH4/CO2_)** | **Y_(x/CO2)_** | **C-balance** |
| --- | --- | --- | --- | --- | --- | --- | --- | --- | --- | --- |
|  |  |  |  |  |  |  |  |  |  |  |
| 0%_1 | -4.69E-02 | -6.84E-02 | 2.14E-02 | 1.33E-02 | 0.7:1 | 5.1:1 | 1.42E-06 | 0.195 | 2.07E-05 | 19.47% |
| 0%_2 | -6.51E-02 | -3.43E-02 | 4.23E-02 | 8.37E-03 | 1.9:1 | 4.1:1 | -6.24E-07 | 0.244 | -1.82E-05 | 24.41% |
| 0%_3 | -6.78E-02 | -3.70E-02 | 4.08E-02 | 1.53E-02 | 1.8:1 | 2.4:1 | 5.67E-07 | 0.413 | 1.53E-05 | 41.32% |
| 0%_4 | -9.25E-03 | -1.30E-02 | 3.46E-02 | 2.74E-03 | 0.7:1 | 4.8:1 | 5.67E-07 | 0.210 | 4.35E-05 | 21.02% |
| 1%_1 | -2.58E+00 | -5.47E-01 | -7.18E-03 | 5.96E-01 | 4.7:1 | 0.9:1 | -6.46E-06 | 1.090 | -1.18E-05 | 109.00% |
| 1%_2 | -2.66E+00 | -5.90E-01 | 2.72E-02 | 6.84E-01 | 4.5:1 | 0.9:1 | -5.33E-06 | 1.159 | -9.03E-06 | 115.91% |
| 1%_3 | -2.49E+00 | -5.76E-01 | 4.60E-02 | 6.80E-01 | 4.3:1 | 0.8:1 | -4.76E-06 | 1.181 | -8.27E-06 | 118.12% |
| 1%_4 | -2.65E+00 | -5.87E-01 | 4.09E-02 | 6.90E-01 | 4.5:1 | 0.9:1 | -1.81E-06 | 1.176 | -3.09E-06 | 117.58% |
| 2.5%_1 | -2.90E+00 | -6.70E-01 | 1.87E-02 | 7.26E-01 | 4.3:1 | 0.9:1 | -1.70E-05 | 1.084 | -2.54E-05 | 108.43% |
| 2.5%_2 | -2.91E+00 | -6.66E-01 | -2.16E-03 | 6.96E-01 | 4.4:1 | 1:1 | 6.69E-06 | 1.046 | 1.01E-05 | 104.59% |
| 2.5%_3 | -3.00E+00 | -6.58E-01 | 1.28E-01 | 8.32E-01 | 4.6:1 | 0.8:1 | 2.21E-06 | 1.264 | 3.36E-06 | 126.42% |
| 2.5%_4 | -4.21E+00 | -6.47E-01 | -2.49E-01 | 5.93E-01 | 6.5:1 | 1.1:1 | 5.54E-05 | 0.916 | 8.55E-05 | 91.63% |
| 5%_1 | -4.31E+00 | -6.20E-01 | -3.84E-01 | 4.12E-01 | 7:1 | 1.5:1 | 8.00E-05 | 0.665 | 1.29E-04 | 66.49% |
| 5%_2 | -4.09E+00 | -6.56E-01 | -2.49E-01 | 5.69E-01 | 6.2:1 | 1.2:1 | 1.37E-04 | 0.867 | 2.09E-04 | 86.70% |
| 5%_3 | -4.22E+00 | -6.14E-01 | -3.50E-01 | 4.29E-01 | 6.9:1 | 1.4:1 | 7.88E-05 | 0.699 | 1.28E-04 | 69.91% |
| 5%_4 | -4.34E+00 | -6.54E-01 | -3.48E-01 | 4.52E-01 | 6.6:1 | 1.4:1 | 6.15E-05 | 0.691 | 9.39E-05 | 69.09% |
| 7.5%_1 | -4.48E+00 | -6.46E-01 | -6.19E-01 | 6.96E-02 | 6.9:1 | 9.3:1 | 9.76E-05 | 0.108 | 1.51E-04 | 10.79% |
| 7.5%_2 | -3.40E+00 | -7.64E-01 | -2.30E-02 | 7.59E-01 | 4.4:1 | 1:1 | 1.49E-05 | 0.993 | 1.95E-05 | 99.29% |
| 7.5%_3 | -3.34E+00 | -7.64E-01 | -5.33E-02 | 7.27E-01 | 4.4:1 | 1.1:1 | 2.45E-05 | 0.951 | 3.21E-05 | 95.13% |
| 7.5%_4 | -4.27E+00 | -6.50E-01 | -3.49E-01 | 4.47E-01 | 6.6:1 | 1.5:1 | 5.78E-05 | 0.688 | 8.89E-05 | 68.84% |
| 10%_1 | -3.22E+00 | -7.61E-01 | -3.20E-02 | 7.43E-01 | 4.2:1 | 1:1 | 1.92E-05 | 0.976 | 2.52E-05 | 97.64% |
| 10%_2 | -4.42E+00 | -6.33E-01 | -4.66E-01 | 2.98E-01 | 7:1 | 2.1:1 | 9.47E-05 | 0.471 | 1.50E-04 | 47.11% |
| 10%_3 | -3.33E+00 | -7.61E-01 | -4.50E-02 | 7.08E-01 | 4.4:1 | 1.1:1 | 2.70E-05 | 0.931 | 3.56E-05 | 93.06% |
| 10%_4 | -3.41E+00 | -7.61E-01 | -6.46E-02 | 7.13E-01 | 4.5:1 | 1.1:1 | 1.81E-05 | 0.938 | 2.39E-05 | 93.80% |
| 50%_1 | -3.64E+00 | -7.57E-01 | -1.53E-01 | 6.11E-01 | 4.8:1 | 1.2:1 | 1.83E-05 | 0.807 | 2.42E-05 | 80.70% |
| 50%_2 | -3.57E+00 | -7.57E-01 | 2.25E-02 | 7.86E-01 | 4.7:1 | 1:1 | 1.72E-05 | 1.038 | 2.28E-05 | 103.78% |
| 50%_3 | -3.48E+00 | -7.57E-01 | -1.04E-01 | 6.62E-01 | 4.6:1 | 1.1:1 | 2.95E-05 | 0.874 | 3.90E-05 | 87.39% |
| 50%_4 | -3.32E+00 | -7.57E-01 | -1.09E-02 | 7.55E-01 | 4.4:1 | 1:1 | 1.01E-05 | 0.997 | 1.33E-05 | 99.66% |
| 100%_1 | -3.33E+00 | -6.96E-01 | -8.94E-02 | 6.21E-01 | 4.8:1 | 1.1:1 | 2.98E-05 | 0.892 | 4.28E-05 | 89.18% |
| 100%_2 | -3.56E+00 | -7.61E-01 | -1.30E-01 | 6.40E-01 | 4.7:1 | 1.2:1 | 2.17E-05 | 0.842 | 2.86E-05 | 84.16% |
| 100%_3 | -3.52E+00 | -7.64E-01 | -2.91E-02 | 7.62E-01 | 4.6:1 | 1:1 | 2.34E-05 | 0.997 | 3.07E-05 | 99.71% |
| 100%_4 | -3.46E+00 | -6.92E-01 | -1.70E-02 | 7.78E-01 | 5:1 | 0.9:1 | 3.06E-05 | 1.124 | 4.42E-05 | 112.40% |

^+^Uptake of gases is visible with negative values. Formation products is visible with positive values. GC analyses were performed after THp_N2fix_ was undercut at 40 h (yellow), 77 h (green) and endpoint at 141 h (blue) of the N_2_-fixation experiment.
